# Real-World Effects of Chinese Herbal Medicine for Idiopathic Membranous Nephropathy (REACH-MN): Protocol of a Registry-Based Cohort Study

**DOI:** 10.3389/fphar.2021.760482

**Published:** 2022-01-03

**Authors:** Lihong Yang, Xueyin Chen, Chuang Li, Peng Xu, Wei Mao, Xing Liang, Qi Zuo, Weizhong Ma, Xinfeng Guo, Kun Bao

**Affiliations:** ^1^ Evidence Based Medicine and Clinical Research Service Group, Guangdong Provincial Hospital of Chinese Medicine/The Second Affiliated Hospital of Guangzhou University of Chinese Medicine, Guangzhou, China; ^2^ Nephrology Department, Guangdong Provincial Hospital of Chinese Medicine, Guangzhou, China; ^3^ State Key Laboratory of Dampness Syndrome of Chinese Medicine, The Second Affiliated Hospital of Guangzhou University of Chinese Medicine, Guangzhou, China; ^4^ Guangdong Provincial Key Laboratory of Chinese Medicine for Prevention and Treatment of Refractory Chronic Disease, The Second Affiliated Hospital of Guangzhou University of Chinese Medicine, Guangzhou, China; ^5^ Guangdong-Hong Kong-Macau Joint Lab on Chinese Medicine and Immune Disease Research, Guangzhou, China

**Keywords:** idiopathic membranous nephropathy, Chinese herbal medicine, cohort study, protocol, registry

## Abstract

**Introduction:** Some encouraging findings of Chinese herbal medicine (CHM) in management of idiopathic membranous nephropathy (IMN) obtained in the setting of clinical trials are hard to validate in the daily clinical practice due to a complicated treatment scenario of CHM in practice. The primary objective of this registry is to provide a description of treatment patterns used in management of IMN and assess clinical remission in daily practice in a Chinese population sample with IMN.

**Methods and analysis:** This is a prospective, multicenter cohort which will comprise 2000 adults with IMN regardless of urinary protein levels that will be recruited from 11 nephrology centers across China. The participants will be followed for up to at least 2 years. Primary outcome is composite remission (either complete remission or partial remission) 24 months after enrolment. The secondary outcomes are complete remission, partial remission, time to remission, no response, relapse, proteinuria, annual change of glomerular filtration rate, antibodies against PLA2R, and composite endpoint of 40% reduction of glomerular filtration rate, doubling of serum creatinine, end-stage renal disease, and death. Propensity score analysis will be used for matching and adjustment.

**Ethics and dissemination:** This study has been approved by the Ethics Committee of Guangdong Provincial Hospital of Chinese Medicine (BF2020-094-01). Results of the study will be published in both national and international peer-reviewed journals, and presented at scientific conferences. Investigators will inform the participants as well as other IMN patients of the findings via health education.

**Study registration**: ChiCTR2000033680 (prospectively registered).

## Introduction

Membranous nephropathy (MN) is an autoimmune disease that affects the kidney glomerulus. It is the leading cause of nephrotic syndrome in nondiabetic adults, counting for 20–37% in most ages and up to 40% in patients over 60 (Ronco and Debiec, 2015; Couser, 2017). The incidence of MN is 10–12 per million persons per year in Europe and the USA, (Maisonneuve et al., 2000), and a rising incidence has been reported in China (Zhang et al., 2017). Approximately 80% of the cases are idiopathic and 20% of them are pathogenically associated with other systematic diseases (i.e., secondary MN). Optimal supportive care and therapy are applied to all patients with idiopathic MN (IMN) (Floege et al., 2019). Immunosuppressive therapy is recommended for IMN patients at risk for progressive kidney injury (e.g. decreasing glomerular filtration rate [GFR]) or with severe life-threatening nephrotic syndrome (Floege et al., 2019). Glucocorticoids combined with cyclophosphamide or calcineurin inhibitors, as well as rituximab, are the major treatment regimens for IMN patients at different risks. They are effective and also accompanied with risks including toxic effects and a high incidence of relapse. For rituximab, the high cost is a large obstacle to acceptability for patients. Other medicines, such as botanicals which include Chinese herbal medicine (CHM), are also widely used in treating IMN in China (Xu et al., 2015).

Studies showed that CHM could be an alternative monotherapy or adjunctive therapy for adults with IMN. Two case reports suggested that 1 year treatment of the herb *Astragalus membranaceus* Fisch. ex Bunge alone was associated with complete remission in patients who were presenting with nephrotic syndrome and resistant to cyclosporine and mycophenolate (Ahmed et al., 2007; Leehey et al., 2010). A retrospective case series comprised of 15 IMN cases who did not respond to immunosuppressive therapies received 6 months treatment of Jian Pi Qu Shi formula, and 80% of them reached complete or partial remission on the 12th month after treatment (Shi et al., 2018). Chen et al. reported a randomized controlled trial that compared multi-ingredient CHM to conventional immunosuppression with prednisone and cyclophosphamide for IMN patients with nephrotic proteinuria (urinary protein excretion ≥3.5g/24h) (Chen et al., 2013). Composite remission (either complete or partial remission) showed a similar rate in the two arms at 48 weeks (73% vs. 78%) (Chen et al., 2013). Liu et al. compared the remission between IMN patients who received tripterygium glycosides tablets (compound of the herb *Tripterygium wilfordii* Hook.f.) plus prednisone and who received tacrolimus plus prednisone (Liu et al., 2015). Both composite remission (86.9% vs. 90.0%) and complete remission (52.2% vs. 46.7%) were similar between the two groups at 36 weeks (Liu et al., 2015).

These findings were obtained in the setting of clinical trials and need validation in daily clinical practice, as the treatment scenario of CHM is more complicated in practice than that in trials. Instead of a fixed formula or proprietary CHM used through the whole research period in clinical trials, prescriptions of CHM are individualized and changed flexibly based on syndrome differentiation or state of the illness in clinical practice. Therefore, we proposed the first registry-based cohort study of INM in China.

The aims of this registry-based cohort include (but are not limited to):

### The Primary Aim

• To summarize the treatment patterns in daily practice for IMN patients in China, and to evaluate the effectiveness in terms of clinical remission among IMN patients who receive different treatment regimens, including CHM, conventional drugs, as well as the combination of both therapies.

### The Secondary Aims

• To identify the potential biomarkers for IMN population and/or subgroup population with specific characteristics.
• To investigate the relationship between population characteristics including Chinese medicine syndromes and tongue features in IMN patients and clinical outcomes.

## Materials and Methods

### Study Design

This is a multicenter prospective hospital-based cohort study, and has been registered with the Chinese clinical trial registry. (ChiCTR2000033680).

### Study Setting

In order to collect a representative sample, the nephrology departments in 11 tertiary-care hospitals across multiple regions in China will recruit and enroll patients continuously. The 11 study sites include Guangdong Provincial Hospital of Chinese Medicine, Guangdong Provincial Hospital of Integrative Medicine, The First People’s Hospital of Zhaoqing, Liuzhou Hospital of Chinese Medicine, and Yue Bei People’s Hospital in south China; Hubei Provincial Hospital of Traditional Chinese Medicine in southwest China; Jiangsu Provincial Hospital of Chinese Medicine and the First Affiliated Hospital of Anhui University of Chinese Medicine in east China; Heilongjiang Institute of Traditional Chinese Medicine and First Affiliated Hospital of Heilongjiang University of Chinese Medicine in northeast China; Shanxi Traditional Chinese Medicine Hospital in northwest China.

### Eligibility Criteria

Eligible candidates for the study are IMN patients of any risk levels to capture a broad spectrum of clinical presentations. Inclusion criteria include Chinese adults aged from 18 to 85 years old; diagnosis of biopsy-proven IMN or antibodies against phospholipase A2 receptor (PLA2R)-positive patients who are not associated with other diseases which cause secondary MN, such as systemic lupus erythematous, infections with hepatitis B virus, hepatitis C virus, or syphilis, sarcoidosis, and cancer. Patients with the following criteria will be excluded: patients with a GFR less than 15 ml/min/1.73 m^2^; patients with comorbidities including malignant cancers, decompensated cirrhosis, hematologic diseases, and mental diseases.

### Interventions

This observational study is designed to evaluate the effectiveness of CHM in a real-word hospital setting. Therefore, there are neither treatment protocols nor interference with therapeutic schedules for the included participants. But the information of therapeutic regimens will be collected to identify the exposure of CHM. Exposure of CHM includes either oral or intravenous administration proprietary CHM products and oral administration CHM formulas. Low, moderate, and high level of CHM exposure will be defined as the cumulative exposure time of CHM accounts for 25, 50, and 75% of individual follow-up time. The individual follow-up time will be from the baseline visit to the last visit or the end of the study.

### Enrollment and Follow-Up

This study is designed to be a long-term longitudinally extensible cohort. The study aims to include 2000 Chinese IMN adults. The recruitment is estimated to take 42 months, from July 2020 until December 2023. The patients who meet the inclusion criteria will be enrolled continuously. Both incident patients and prevalent patients will be recruited by physicians in nephrology clinics and inpatient departments in 11 participating sites. Incident patients are individuals who are newly diagnosed and first initiated on treatments. Prevalent patients are individuals who have already been diagnosed as IMN and have been receiving treatments before the study. Meanwhile, the electronic medical record in 11 participating sites will be retrospectively screened to recruit the confirmed cases.

**FIGURE 1:**
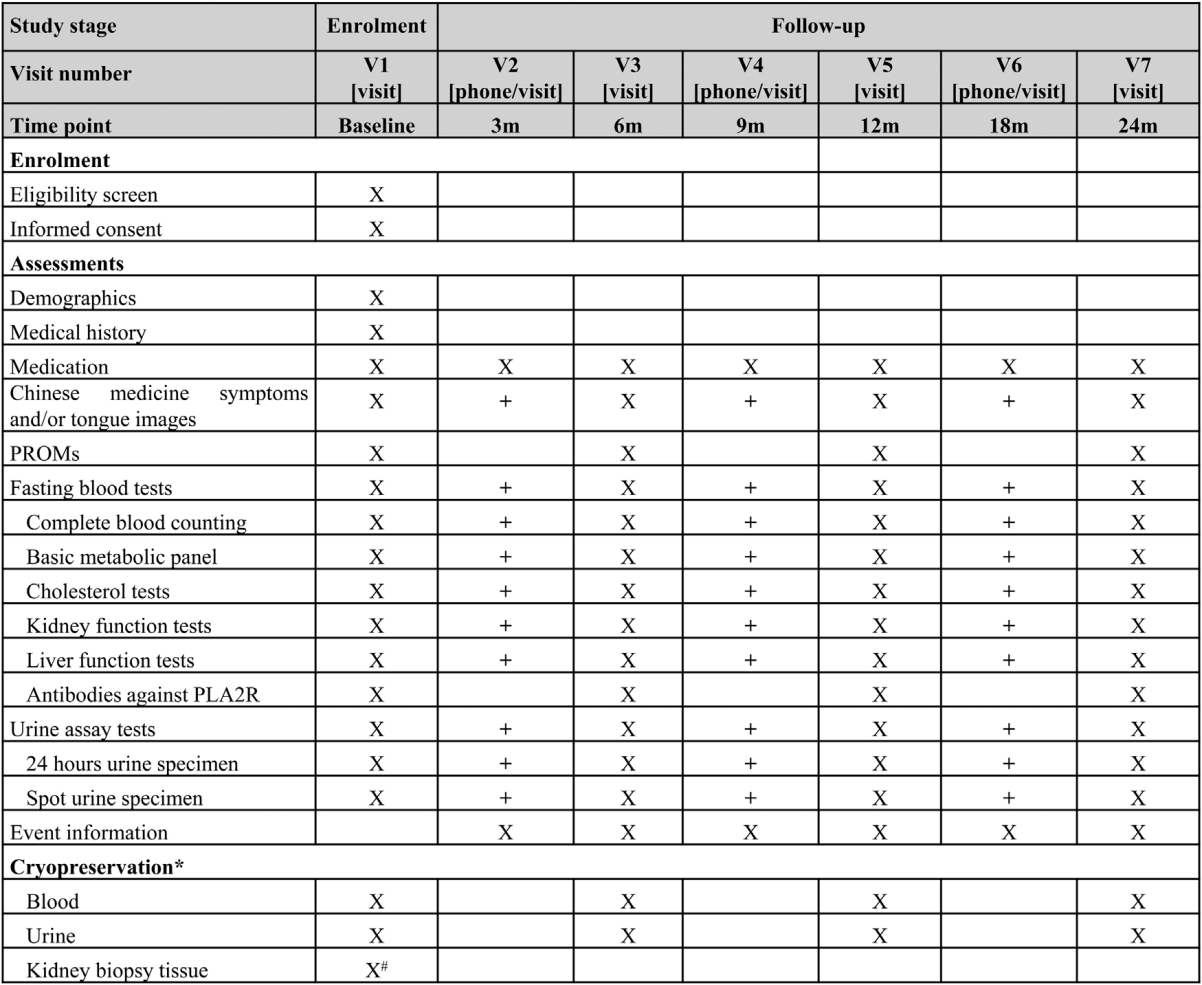
Visit schedule of enrollment and follow up; * Only collected in the centers where specimen storage is available. # Not required for the prevalent patients X Required. + Optional. RPOMs: patient reported outcome measures, PLA2R: phospholipase A2 receptor.

Individuals who are eligible and consent to participate will be invited to attend a baseline visit. Participants will be followed for up to at least 2 years. Participants who experience the primary endpoint (remission) will be continued to be followed up until the end of the study or death. The schedule of follow-up visits is presented in Figure 1. Follow-up visits will occur quarterly in the first year after enrollment and then semi-annually from the second year. The baseline, semi-annual, and annual follow-up visits in the first year and annual visits from the second year are required in-person visits. All other visits can be in-person or remote via telephone call or smartphone applications. In order to retain participants and minimize the number of dropouts and withdraws as well as the missing data, a reasonably flexible visit time window within a range of 4 weeks forward or backward from the scheduled visit day will be acceptable.

### Data Collection and Management

The data elements of the personal domain (demographics, medical history, Chinese medicine specific information including symptoms and tongue images, etc.), the exposure domain (treatment prescriptions of CHM and conventional drugs), and the outcome domain will be recorded. Each participant will have a unique number, and their personally identifiable information will not be collected. Both spot urine specimen and 24 h urine specimen will be collected to assay for red blood cells, white blood cells, urinary protein-to-creatinine ratio, and 24 h total urinary protein. Fasting blood draw will be tested for complete blood cells (red blood cell, hemoglobin, white blood cell, neutrophil, leukomonocyte, and platelets), metabolic panel (blood glucose, potassium, albumin, aspartate aminotransferase, alanine aminotransferase, uric acid, creatinine, total cholesterol, triglycerides, and low-density lipoprotein), and PLA2R. GFR will be estimated using the 2009 Chronic Kidney disease Epidemiology Collaboration (CKD-EPI) equation based on serum creatinine (Levey et al., 2009). Recently, a new equation based on both serum creatinine and cystatin C, which is also proposed by CKD-EPI, will be used to estimate GFR as well (Inker et al., 2021). Patient report outcome measures including the Kidney disease Quality of Life-36 which will be used to assess quality of life. At the baseline and each annual visit, a blood sample and a urine sample will be collected for cryopreservation, which will be used for detection of disease specific biomarkers in further research phases. Additionally, if participants are newly diagnosed by kidney biopsy, the kidney biopsy tissue will be cryopreserved as well. The scheduled time points for data collection are summarised in Figure 1.

**FIGURE 2:**
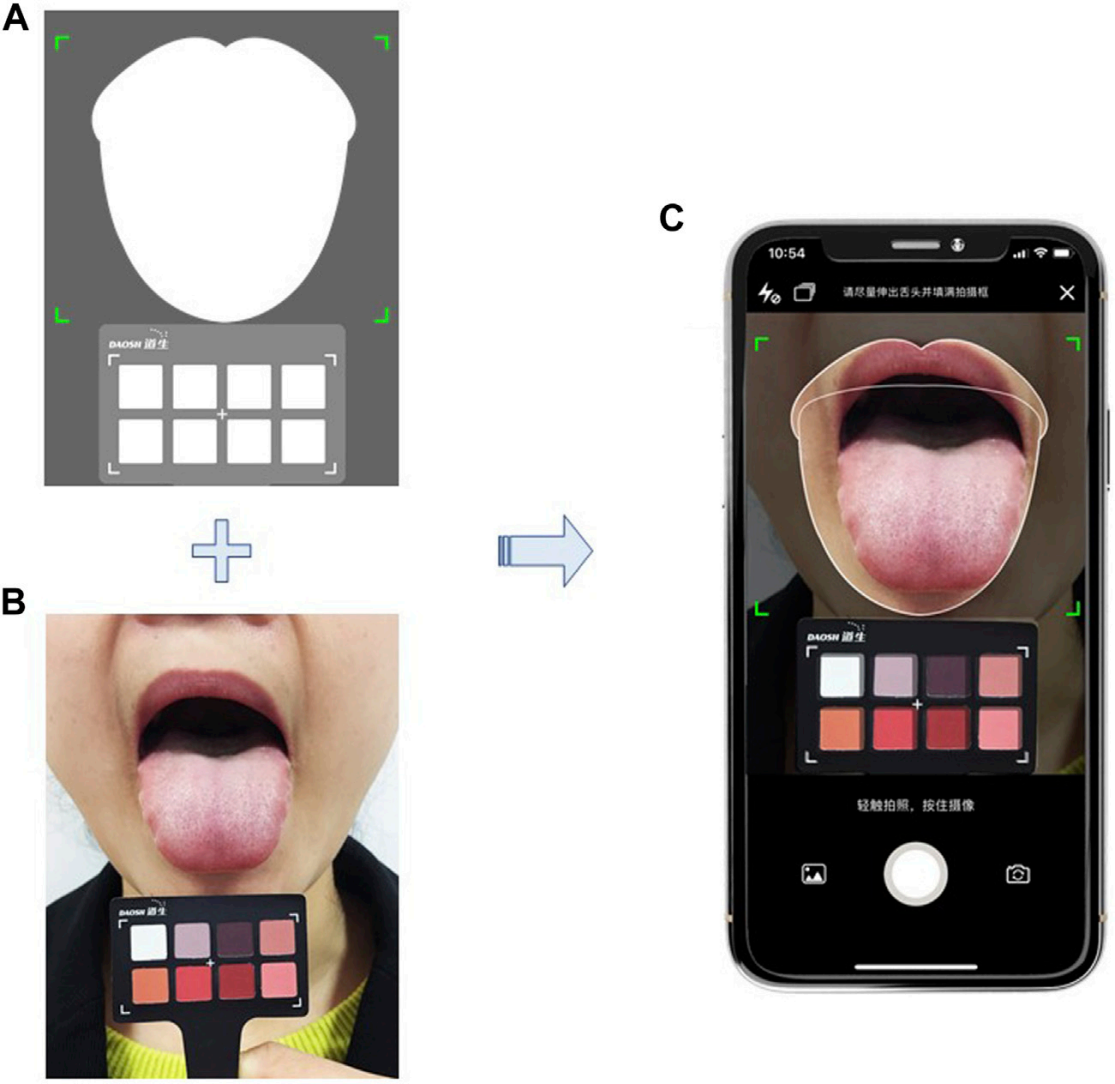
Tongue image capture by using a smart phone application; includes a demonstration of training; **(A)** interface of the tongue image acquisition application, including a tongue image area and a color correction chart area; **(B)** standardized operation for sticking tongue out and position of a standard color correction chart; **(C)** take photo with the tongue image acquisition application in a smart phone. *Note: The person depicted is not a patient and was taken with the participant’s knowledge.

The data of the core Chinese medicine syndrome of dampness in IMN will be collected by using the validated Dampness Syndrome Scale (Lu et al., 2021) and the tongue image acquisition system. The scale consists of 30 items. There are five options for each item from 0 to 4 which indicate no symptoms, mild, moderate, heavy, and very heavy symptoms. The total score ranges from 0 to 120. More than 15 items with at least one point can be defined as dampness syndrome. The higher the score, the more severe the dampness syndrome. Tongue images will be captured by using a smartphone tongue image acquisition application with a standard color correction chart (Figure 2) and quantified the tongue characteristic by using a computerized tongue image analysis system (Shanghai Daosh Medical Technology Co., Ltd.). (ISO, 2019; ISO/TS, 2020).

All data will be collected and recorded using electronic data collection (EDC). A systematic training is required for the personnel in all participating sites before the commencement of recruitment.

### Outcomes

The primary outcome is clinical composite remission at 24 months after enrollment. Composite remission is defined as either complete remission (CR) or partial remission (PR). Secondary outcomes are CR, PR, time to remission (CR, PR, and CR or PR), no response, relapse, urine protein excretion, urinary protein creatinine ratio, serum albumin, annual change of GFR (GFR slope), PLA2R, adverse events, and composite endpoint of 40% reduction of GFR, doubling of serum creatinine, end-stage renal disease (ESRD), and death. The outcome variables and definitions are presented in Table 1.

**TABLE 1:** Outcome variables and definitions.

Outcome variables	Definitions
Primary outcome	
Composite remission	Either complete remission or partial remission
Secondary outcomes	
Complete remission (CR)	A reduction of proteinuria to ≤0.3g/24 h accompanied by a normal serum albumin and a normal Scr. Beck et al. (2013)
Partial remission (PR)	At least 50% reduction from peak value of proteinuria to a level between 0.3 and 3.5 g/24 h, accompanied by an improvement or normalization of serum albumin and a stable Scr. Beck et al. (2013)
Relapse	The development of proteinuria ≥3.5 g/24 h after a CR or PR
Time to complete remission	Days from enrollment to CR
Time to partial remission	Days from enrollment to PR
No response	No reduction in proteinuria of at least 25% from baseline
Serum albumin	/
Urine protein excretion	Urine protein excretion within 24 h
Urinary protein-to-creatinine ratio (UPCR)	/
Antibodies against PLA2R	/
Doubling of serum creatinine	Doubling increase of serum creatinine from baseline
40% reduction of eGFR	A reduction in eGFR of 40% from baseline. Serum creatinine based eGFR is calculated using the Chronic Kidney disease Epidemiology Collaboration (CKD-EPI) equation
End-stage renal disease (ESRD)	eGFR ≤15 ml/min/1.73m^2^ or commencement of renal replacement treatment
Composite endpoint	ESRD or 40% reduction of eGFR or doubling of serum creatinine
eGFR slope	Annual change in eGFR

### Sample Size

The composite remission rate in Chinese adults with IMN under treatment of immunosuppressive agents in clinical practice in a single center in China was 73.7% (Jiang et al., 2021). An analysis of our retrospective unpublished data from three hospitals in China showed that composite remission rate of IMN patients received CHM alone or combination therapies in clinical practice was 67.0% (75/112). Under the assumption of 10% of loss to follow-up and two-sided alpha level of 0.05, enrolment of 2000 patients would provide 80% power to detect the 2-year composite remission rate.

### Statistical Plan

Attempting to ensure equivalence in baseline characteristics of participants between the CHM group and the conventional therapy group, a propensity score method will be performed. Variables involving age, sex, proteinuria, eGFR, serum albumin, uric acid, PLA2R antibody level, and pathological type will be included into the logistic regression model to calculate a propensity score for each participant. Participants who are exposed to CHM will be matched to those who receive conventional drugs alone based on the propensity score, using the greedy matching with a standard calliper width of 0.2 and without case replacement. Covariate balance of the matched cohort will be assessed using the mean standardized differences. When a |d| > 0.10, it is considered as an imbalance. Inverse probability of treatment weighting based on the propensity score will also be performed to evaluate the robustness of the matching results.

Baseline demographic variables will be presented as means, medians, or percentages, and using Chi-square test, independent t test, or Mann-Whitney *U* test, as appropriate. Outcomes of composite remission, CR, PR, no response, death, relapse, and composite endpoint will be analysed by using Chi-square test and logistic regression. Odd ratios and 95% confidence intervals will be estimated. Time to remission will be analysed by using Cox regression model, adjusting for gender, age, baseline proteinuria, GFR, PLA2R antibody level, uric acid, Chinese medicine syndrome, and propensity score.

Subgroup analysis will be performed on age, gender, diagnosis criteria (i.e., biopsy proven or anti-PLA2R positive), PLA2R positive or negative, blood lipid levels, different risk levels of progressive loss of kidney function, “incident patient” and “prevalent patient”, Chinese medicine syndromes, different assay methods for serum creatinine and proteinuria, different levels of CHM exposure, treatment regimens (CHM alone, conventional medication alone and the combination), conventional medications, and CHM with and without immunosuppressive effect. For the analyses of Chinese medicine syndromes, besides Chi-square test between groups of patients with and without dampness syndrome, a trend test will be performed to assess the dose-response relationship between dampness syndrome and clinical outcomes. CHM with immunosuppressive effect will be defined as either herbal formulas or proprietary CHM products which contain either *Tripterygium wilfordii* Hook. f, *Tripterygium hypoglaucum* (Levl.) Hutch and *Sinomenium acutum* (Thumb.) Rehd. et Wils (Menispermaceae, SA) or their compounds (Zhao et al., 2012; Chen et al., 2014; Pei et al., 2016; Lv et al., 2019; Zheng et al., 2019).

GFR will be estimated by 2009 CKD-EPI creatinine equation and 2021 CKD-EPI creatinine-cystatin C equation. As the 2021 equation has not yet been validated in the Chinese population, analyses involved eGFR will use the estimates calculated by the 2009 equation, and sensitivity analyses based on the 2021 equation will be performed. To eliminate the acute effect, incident patients who experience a serum creatinine increase of more than 30% within 12 weeks after initiated treatments will be excluded in analyses on endpoints in terms of ESRD, 40% eGFR reduction, and eGFR slope.

Additionally, in order to assess the risk of performance bias, the change of prescription pattern before and after the commencement of the study will be analyzed.

All analysis will be tested with a two-sided alpha level of 0.05. For the missing data, the reasons why the data is missing will be analyzed, and multiple imputation will be used.

### Ethics and Dissemination

This study has been approved by the Ethics Committee of Guangdong Provincial Hospital of Chinese Medicine (BF 2020-094-01) on June 3, 2020. A copy of ethical approval documents will be provided to the local research ethics boards of each participating site. The study team will follow the study standard operating procedures to ensure that all participants’ consent will be informed and voluntary. The participants have the right to withdraw at any time, and they will be given a copy of signed informed consent forms.

As this is an observational study, there are no financial and other competing interests for principal investigators for the whole study and each participating site, as well as the funding agencies.

Important protocol modifications, such as, changes to eligibility criteria, outcomes, and participating sites, will be submitted to the Ethics Committee of Guangdong Provincial Hospital of Chinese Medicine for approval. An updated version will upload into the Chinese clinical trial registry after the approval.

Results of the study will be published in both national and international peer-reviewed journals and presented at scientific conferences. Investigators will inform the participants as well as other IMN patients of the findings via health education.

## Discussion

This national-wide, prospective, observational registry provides a platform for multi-center longitudinal research in IMN. During the first 5-year research phase of the registry, it is expected to provide a description of treatment patterns in patients with IMN in China, and more than that, to provide an estimation of the clinical remission of this population undergoing various therapeutic regimens in daily practice.

Quality control is vital in clinical studies, especially in multicenter studies. Our multicenter registry uses EDC to collect data and promote the quality of the data. Instead of finding errors after the data collection period, EDC allows researchers to build logical checks and pre-fillable answers, which can reduce the number of errors in the data at the collection site. Further, EDC keeps all traces of operation to ensure the authenticity of data.

Several bias may occur in a cohort. The nature of the cohort study could induce the participant selection bias, such as referral bias. As all 11 centers that participated in the study are tertiary-care hospitals, patients recruited from the centers with a particular severity differ from those in the community or general population, especially among prevalent patients (i.e., individuals who have already been receiving treatments before enrollment). Considering the incidence of IMN and the availability of subjects, in order to recruit enough samples on time, our cohort will include both incident patients who are newly diagnosed and first initiated on treatments and prevalent patients. Subgroup analysis of incident patients and prevalent patients will be performed to investigate whether the results are robust among the whole sample and different risk of subgroups and overcome the referral bias. In addition, in order to minimize the risk of selection bias, a propensity score approach will be used to balance the baseline characteristics of the CHM group and conventional group.

Another potential bias is performance bias. The study aims to observe the remission under the treatment of CHM and conventional therapies in clinical practice without any interference to the treatment regimens. The study is a registry-based cohort and blinding is not involved. Researchers may change their treatment strategies on purpose to align more with clinical practice guidelines. The research committee will emphasize that researchers in all centers are going to treat the included patients as usual and do not change the treatments purposefully. Further, a comparison of the prescription pattern before and after the start of the study will be used to assess the risk of performance bias.

As the study is based on a long-term longitudinally extensible registry, it will take several years to reach the target of the full construct. Therefore, retention of the participants is a key to success and a challenge. The burden of both financial and time consuming for participants in our study is low. First, there is no extra cost. Besides, patients will get a reimbursement for transportation in each in-person visit. Second, times of in-person visits are minimized to once or twice a year and the time window of visits is relatively flexible. Moreover, the participants will be under intensive health monitoring.
